# Cognitive impairment in adolescent and young adult cancer patients: Pre‐treatment findings of a longitudinal study

**DOI:** 10.1002/cam4.5295

**Published:** 2022-10-11

**Authors:** Alexandre Chan, Ivy Cheng, Claire Wang, Chia Jie Tan, Yi Long Toh, Ding Quan Ng, Yong Qin Koh, Hanzhang Zhou, Koon Mian Foo, Raymond Javan Chan, Han Kiat Ho, Lita Chew, Mohamad Farid, Ian Tannock

**Affiliations:** ^1^ Department of Clinical Pharmacy Practice University of California Irvine Irvine California USA; ^2^ Department of Pharmacy National Cancer Centre Singapore Singapore; ^3^ Department of Pharmacy National University of Singapore Singapore; ^4^ Department of Pharmacy KK Women and Children's Hospital Singapore; ^5^ Caring Futures Institutes, College of Nursing and Health Sciences Flinders University Adelaide South Australia Australia; ^6^ Division of Medical Oncology National Cancer Centre Singapore Singapore; ^7^ Princess Margaret Cancer Centre Toronto Ontario Canada

**Keywords:** adolescent and young adult, brain‐derived neurotrophic factor, cancer, cancer‐related cognitive impairment, cognition, inflammatory cytokines

## Abstract

**Background:**

There is little information about cancer‐related cognitive impairment (CRCI) in adolescent and young adults (AYA, 15–39 years old) due to its rare incidence. Here, we present the pre‐treatment (before chemotherapy or radiotherapy) evaluation of cognitive function and ability of AYA with cancer (AYAC) in a multicentered cohort study.

**Methods:**

Newly diagnosed AYAC and age‐matched healthy controls (HC) were recruited between 2018 and 2021. The primary outcome was the comparison of pre‐treatment cognitive impairment defined as 2 standard deviations (SDs) below the HC on ≥1 cognitive test, or >1.5 SDs below on ≥2 tests using CANTAB® between AYAC and HC. Secondary outcomes included self‐perceived cognitive ability assessed by FACT‐Cog v3 and biomarkers (inflammatory cytokines and brain‐derived neurotrophic factor [BDNF]).

**Results:**

We recruited 74 AYAC (median age = 34) and 118 HC (median age = 32). On objective cognitive testing, we observed three times more AYAC patients performed poorly on at least 2 cognitive tests compared to HC (40.5% vs. 13.6%, *p* < 0.001). AYAC self‐perceived less degree of cognitive impairment than HC (*p* < 0.001). However, AYAC perceived a greater impact of cognitive changes on their quality of life compared to HC (*p* = 0.039). Elevated baseline inflammatory markers (IL‐2, IL‐4, IL‐6, IL‐8, IL‐10 and IFN‐γ) were observed among AYAC compared to HC, and baseline BDNF was lower in AYAC compared to HC. Interaction effects between cancer diagnosis and biomarkers were observed in predicting cognitive function.

**Conclusion:**

With the pre‐existence of CRCI and risk factors of neuroinflammation even prior to systemic therapy, AYAC should receive early rehabilitation to prevent further deterioration of cognitive function after initiation of systemic therapies. (ClinicalTrials.gov Identifier: NCT03476070).

## Graphical abstract

This study reported about pre‐treatment cancer‐related cognitive impairment between newly diagnosed AYAC and age‐matched healthy control. There were higher proportions of subjects with impairment and elevated inflammatory marker levels among AYAC compared to controls. At the same time, lower levels of self‐perceived impairment and lower baseline BDNF levels were reported in this patient group.

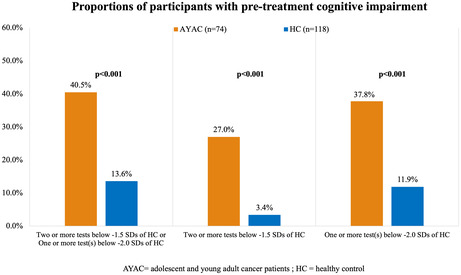

## 1 BACKGROUND

An Adolescent and Young Adult cancer patient (AYAC) is defined as an individual 15 to 39 years of age at the time of initial cancer diagnosis.^1^, ^2^ Diagnosis of invasive cancer is rare within this age range.^3^ Due to the rarity of cancer incidences, it is difficult to conduct research to study survivorship issues in this unique group of cancer survivors. Unfortunately, these cancer survivors often experience treatment‐related chronic and late toxicities that can lead to functional impairment at great economic, emotional, and social cost.^4^ As the cure rates for AYA cancers continue to improve and survivors live longer, their post‐treatment health issues, such as cognitive toxicity, are becoming increasingly relevant, and more in‐depth research is needed.^3^, ^4^, ^5^, ^6^, ^7^ Our preliminary data suggested that one in three AYAC self‐perceived cognitive impairment within the first year post chemotherapy.^8^ It is important to recognize cognitive toxicity among AYAC, and to study underlying mechanisms in order to provide age‐appropriate care and rehabilitation.^9^, ^10^

Although much of the literature suggests that cognitive toxicity is due to the treatment that the patients receive (hence the term ‘chemobrain’), several studies have suggested that impaired cognitive performance exists among older patients with cancer, prior to the initiation of treatment. For example, higher proportions of women with breast cancer (median age = 55 years old) reported self‐perceived cognitive impairment as compared with age‐matched controls prior to chemotherapy.^11^ Similarly, a large observational study of colorectal cancer and healthy controls has shown that patients had slower processing speed, working memory problems and verbal learning inefficiency prior to chemotherapy.^12^ Neuroimaging studies have also shown that brain activation due to high demand tasks involving attention and working memory were more common among older women with breast cancer who were undergoing fMRI‐related tasks prior to chemotherapy compared to their non‐cancer counterparts.^13^

Previous studies of AYAC have not compared their performance to that of healthy controls or examined cognitive function prior to cancer treatment.^8^, ^14^ Here we report the prevalence of pre‐treatment cognitive impairment in AYAC compared to non‐cancer controls. The primary objective of this study is to compare pre‐treatment cognitive function using evaluation with the Cambridge Neuropsychological Test Automated Battery (CANTAB®) between AYAC and healthy controls (HC). Secondary outcome measures included differences between AYAC and HC on self‐perceived cognitive ability assessed by FACT‐Cog v3, health‐related quality of life, cancer‐related fatigue, and biomarkers (inflammatory cytokines and brain‐derived neurotrophic factor).

## 2 METHODS

### 2.1 Study design

This is a cross‐sectional analysis of the Adolescent and Young Adult Cancer Patients: Cognitive Toxicity on Survivorship (ACTS) study. ACTS was designed as a multicenter, prospective, longitudinal, observational study conducted at the National University of Singapore, National Cancer Centre Singapore and KK Women's and Children's Hospital between June 2018 and December 2021. Over 70% of all AYAC in Singapore receive ambulatory cancer care from NCCS and KKH. The study protocol received ethics approval from the Singhealth Institutional Review Board (CIRB 2017/3139) and all study participants provided written informed consent prior to participation. (Clinicaltrials.gov: NCT03476070).

### 2.2 Inclusion/Exclusion criteria

Two groups of participants were recruited for this study: AYAC and age‐matched HC.

#### 2.2.1 AYAC

Participants were between 15 and 39 years of age, newly diagnosed with cancer and seeking care in the outpatient setting. They must not have received chemotherapy or radiotherapy and were capable of giving informed consent (and obtaining parents' consent together, if required). Those with evidence of psychosis or underlying neuropsychiatric illness that might impair cognitive abilities, were excluded from the study.

#### 2.2.2 HC group

Volunteers were between 15 and 39 years of age and capable of giving informed consent. The same eligibility criteria (except the cancer diagnosis) applied to HC. They were community controls and recruited through advertisement, word of mouth and patient referral.

### 2.3 Data collection

Recruitment occurred immediately after diagnosis of cancer by medical oncologists, and study assessments were conducted within appointments before patients received prescribed treatments. All questionnaires were administered in person by a trained research assistant in English. All participants completed study questionnaires including the Functional Assessment of Cancer Therapy – Cognitive Function (FACT‐Cog), the Rotterdam Symptom Checklist (RSCL), Pediatric Quality of Life Inventory (PedsQL™) and the Multidimensional Fatigue symptom Inventory‐Short Form (MFSI‐SF). Participants completed a battery of neuropsychological tests using the Cambridge Neuropsychological Test Automated Battery (CANTAB) to assess objective cognitive function. Relevant demographic and clinical data were also collected through patient interviews and electronic health records.

1. *CANTAB*: Objective cognitive function was evaluated by CANTAB on a tablet computer across five tasks in the following order: multitasking test (MTT), paired associates learning (PAL), reaction time (RTI), spatial working memory (SWM), and rapid visual information processing (RVP). These measures correspond to the domains of multitasking, memory, response, executive function, and attention, respectively. Better cognitive performance was indicated by higher values on RVP (better able to detect target sequences), as well as lower values on RTI (faster reaction times), PAL (less errors), SWM (more strategy use), and MTT (less multitasking cost). A description of these measures can be found in Data S1.
2. The *FACT‐Cog (Version 3)* is a validated questionnaire used to assess perceptions of quality of life and impact of cognitive abilities in cancer populations within the past 7 days. The questionnaire comprises 37 items scored on a five‐point Likert scale, from 0 (“Never” or “Not at all”) to 4 (“Several times a day” or “Very much”). The items form four sub‐scales: perceived cognitive impairment (PCI; 20 items, score range 0 to 80), comments from others (OTH; 4 items, score range 0 to 16), perceived cognitive abilities (PCA; 9 items, score range 0 to 36) and impact on quality of life (QOL; 4 items, score range 0 to 16). Negatively worded items were reverse scored. Scores were summed for each subscale, with higher values indicating better subjective cognitive function.
3. The *RSCL* evaluates symptoms reported by cancer patients, and covers 4 domains: physical symptom distress (23 item), psychological distress (7 item), activity level (8 item), and overall global life quality (single item).^15^ Each response is on a 4‐point Likert scale. The scores are transformed to a 100‐point scale for comparison using the formula: [(raw score‐minimum raw score)/ (maximum‐minimum score) x 100].
4. The *PedsQL* Version 4.0 is designed to assess health‐related quality of life.^16^ PedsQL was chosen in view that it could span across the AYA age spectrum. Three versions of the generic core scales were used: teens (ages 13–18), young adults (18–25), and adults (age over 26). It consists of 23 items comprising 4 dimensions in physical, emotional, social and work/studies functioning. A psychosocial health summary score (total score 0–100) can be calculated from the sum of the items over the number of items answered in emotional, social and work/studies functioning while a physical health summary score is derived from the physical functioning scale score (total score 0–100).
5. The *MFSI‐SF* questionnaire assesses fatigue in cancer patients.^17^, ^18^ It consists of five subscales with six items each: general fatigue, physical fatigue, emotional fatigue, mental fatigue, and vigor. Each domain is rated on a scale of 0 to 4. The total score is obtained by summing all the dimension except the vigor domain which is subtracted. The total score ranges from −24 to 96, with higher scores indicating more fatigue.

#### 2.3.1 Biomarker analysis

A 9‐ml blood sample was collected from each participant before the administration of chemotherapy and stored in ethylenediaminetetraacetic acid tubes. Sample collection procedures were standardized and previously reported elsewhere.^19^, ^20^ It was centrifuged at 1069 x *g* for 10 min at 4*°*C, and the plasma sample was stored in a −80*°*C freezer until sample analysis. Two types of plasma biomarkers were analyzed:

1. *Inflammatory cytokines* were quantified using 50 μl of each sample with the multiplexed immunoassay (Bioplex Human Cytokine 9‐Plex Panel, Biorad) performed in duplicate. The cytokine panel consisted of interferon (IFN)‐ƴ, tumor‐necrosis factor (TNF‐α), granulocyte‐macrophage colony‐stimulating factor (GM‐CSF), interleukin (IL)‐2, IL‐4, IL‐6, IL‐8, and IL‐10.
2. *Brain‐derived neurotrophic factor (BDNF)*: BDNF levels were quantified using 100 μl of sample diluted 100‐fold using a commercially available enzyme‐linked immunosorbent assay (ELISA) kit (Biosensis BEK‐2211‐1P/2P, Australia) and performed in duplicate. The concentration of BDNF was first calculated in ng/ml with four‐parameter logistic regression followed by transformation to ng/ml.

### 2.4 Endpoints

The primary outcome was the comparison of pre‐treatment cancer‐related cognitive impairment using CANTAB® between AYAC and HC, defined as 2 standard deviations (SDs) below the HC on ≥1 cognitive test, or >1.5 SDs below on ≥2 tests using CANTAB®. Secondary outcomes included self‐perceived cognitive ability assessed by FACT‐Cog v.3 and biomarkers (inflammatory cytokines and BDNF), symptom burden (RSCL), quality of life (PedsQL), and fatigue (MFSI‐SF). Psychological distress and fatigue were determined using the RSCL psychological distress subscale and MFSI‐SF total score, respectively.

### 2.5 Statistical analysis

Categorical data are presented as counts and percentages while continuous data are summarized with means and SDs, or medians and interquartile ranges, depending on skewness. Baseline demographic and clinical parameters were tested for differences between AYAC and HC with Chi‐square test or Fisher's exact test for categorical variables, and t‐test or Mann–Whitney U test for continuous variables. Spearman's rank correlation was conducted to assess the relationship between the domains of objective and subjective cognitive function. Multivariable linear regression adjusting for clinically relevant demographic variables (age, gender, ethnicity, marital status, highest education level, psychological distress, and fatigue) was performed to assess the differences in cognitive outcomes between AYAC and HC. Marital status (married vs never married/divorced/widowed) and education level (undergraduate/post‐graduate vs primary/ secondary/pre‐university) were regrouped as two levels for regression analysis.

To assess the robustness of our primary findings, an unplanned sensitivity analysis was conducted. Propensity score weighting using inverse probability weights was carried out to address imbalances in baseline demographic characteristics between the AYAC and HC groups. Details of the sensitivity analysis are provided in Data S1.

The main effects for biomarkers and their interaction with cancer (cancer vs no cancer) were used to determine the associations between biomarker levels and cognitive outcomes. Linear combinations were evaluated to assess the differences in the biomarker‐cognitive outcomes relationships between AYAC and HC. Associations between biomarkers and cognitive outcomes were conducted after controlling for known to impact cognitive function (age, gender, ethnicity, marital status, highest education level, psychological distress, and fatigue).

All statistical analyses were two‐sided and conducted on R v4.1.2 (on RStudio Build 382), Graphpad Prism version 9 and Stata version 16.1 (College Station, TX). The significance level of tests was not corrected for multiple comparisons and other than the test addressing the primary hypothesis, they are regarded as exploratory and hypothesis generating.

## 3 RESULTS

### 3.1 Demographics and clinical characteristics

**FIGURE 1:**
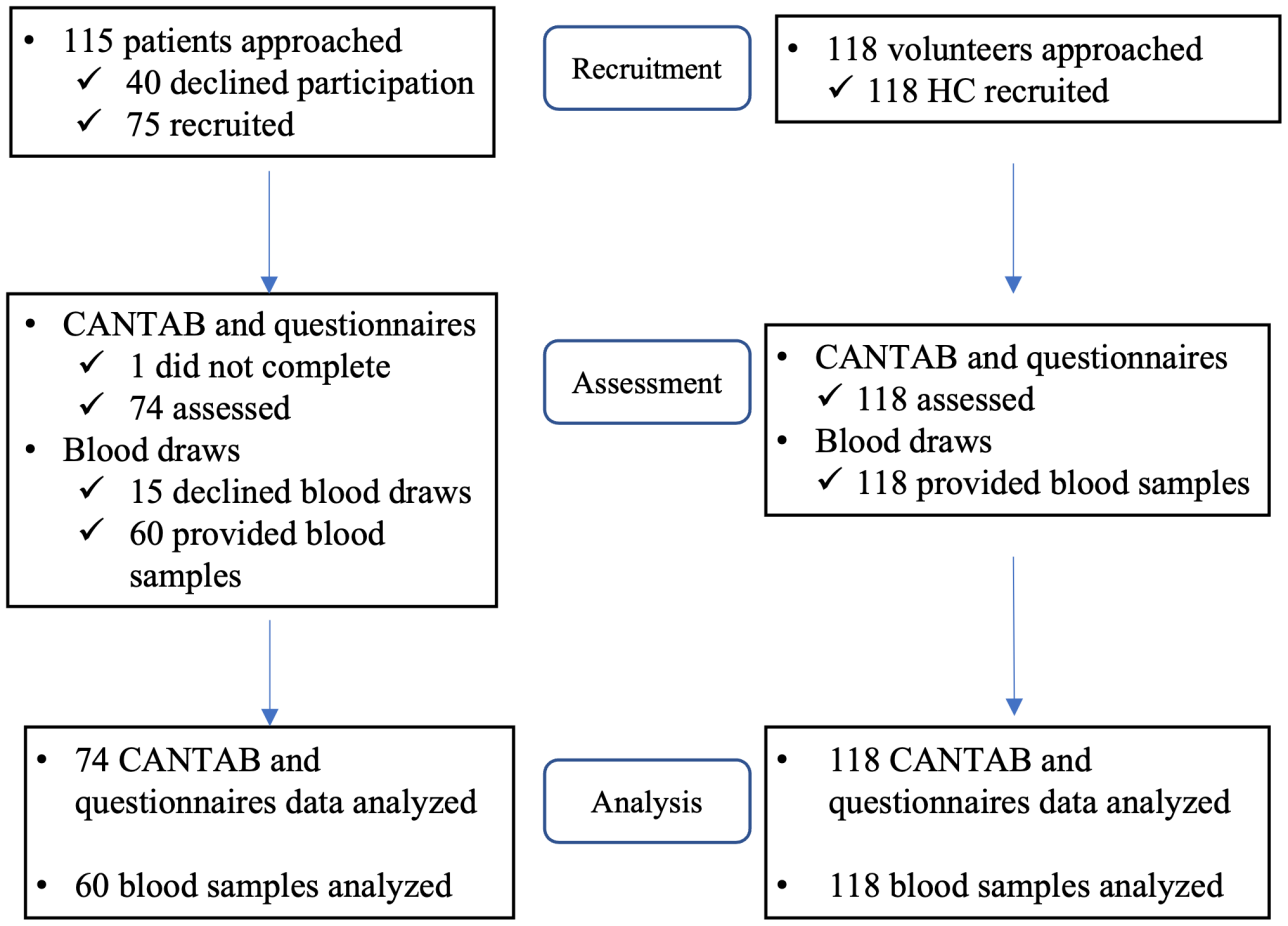
Flow chart of participant recruitment.

Seventy‐four AYAC and 118 HC were recruited and provided analyzable data (Figure 1). Median age and the interquartile range for patients (34 [28–36] years old) and HC (32 [27–34] years old) were similar; both arms recruited mostly female participants (64% for both arms) (Table 1). The ethnic composition of the two arms differed (*p* < 0.001), with a higher proportion of Malay (AYAC: 18%; HC: 2%) and lower proportion of Indian participants (AYAC: 5%; HC: 19%) in the patient arm compared to HC. Compared to HC (41%), a higher proportion of patients (60%) were married (*p* = 0.013), and a higher proportion of HC had completed at least pre‐university education (patients: 76%, HC: 99%, *p* < 0.001). Patients were mostly diagnosed with breast (24%) and head and neck (22%) cancers. Most AYAC had good performance status, with an ECOG status of 0 (88%) and 1 (9%). Planned treatment modalities were chemotherapy (89%), radiotherapy (66%), and surgery (43%) for AYAC. **(**Table 2 **).**

**TABLE 1:** Subject demographics and clinical characteristics

Characteristic	AYAC (*N* = 74)^a^	HC (*N* = 118)	*p*‐value
Demographic characteristics			
Age in years, median (IQR)	34 (29–37)	32 (28–35)	0.06
Gender, *n* (%)			
Male	27 (36%)	43 (36%)	1.00
Female	47 (64%)	75 (64%)	
Ethnicity, *n* (%)			
Chinese	51 (69%)	89 (75%)	< 0.001
Malay	13 (18%)	2 (2%)	
Indian	4 (5%)	22 (19%)	
Others^b^	6 (8%)	5 (4%)	
Marital status, *n* (%)			
Never married	28 (38%)	68 (58%)	0.013
Married	44 (60%)	48 (41%)	
Divorced	2 (3%)	1 (1%)	
Widowed	0	1 (1%)	
Highest education level, *n* (%)			
Primary school	1 (1%)	0	< 0.001
Secondary school	17 (23%)	1 (1%)	
Pre‐university	14 (19%)	13 (11%)	
Undergraduate/Postgraduate	42 (57%)	104 (88%)	
Years of education (median)	15 (12,17)	17 (16,19)	< 0.001

Abbreviations: AYAC, AYA patients with cancer; HC, healthy control.

^a^ 75 patients and 118 controls were recruited for the study. 1 patient's data were excluded from analysis due to missing data.

^b^ Includes 2 Filipinos, 2 Sikhs, 1 Bhutanese and 1 Javanese patients, as well as 4 Filipino and 1 Vietnamese HC.

**TABLE 2:** Clinical characteristics of AYAC (*n* = 74)

Diagnosis, *n* (%)	
Breast	18 (24%)
Head and neck	16 (22%)
Gynecological	14 (19%)
Lymphoma	10 (14%)
Testicular	6 (8%)
Sarcoma	4 (5%)
Lung	2 (3%)
Colorectal	2 (3%)
Thyroid	1 (1%)
Esophageal	1 (1%)
Stage, *n* (%)	
0	1 (1%)
1	12 (16%)
2	24 (32%)
3	20 (27%)
4	13 (18%)
Not indicated	4 (5%)
Baseline ECOG status, *n* (%)	
0	65 (88%)
1	7 (9%)
2	2 (3%)
Planned treatment modality, *n* (%)	
Chemotherapy	66 (89%)
Radiotherapy	49 (66%)
Surgery	32 (43%)
Endocrine therapy	5 (7%)
Immunotherapy	2 (3%)

Abbreviation: AYAC, AYA patients with cancer.

### 3.2 Prevalence of pre‐treatment cognitive impairment

Based on our pre‐defined criterion, we observed a significantly higher (*p* < 0.001) proportion of subjects with impairment among AYAC (40.5%) as compared to HC (13.6%). The distributions among HC are similar to the expected proportions modeled by Ingraham and Aiken^21^ (Table 3).

**TABLE 3:** Objective (CANTAB®) and subjective (FACT‐Cog) cognitive function

Objective cognitive function (CANTAB®)	AYAC (%) (*N* = 74)	HC (%) (*N* = 118)	*p*‐values	Expected proportion^@^ (%)
**≥**2 cognitive tests below −1.5 SDs of HC or **≥**1 cognitive test(s) below −2.0 SDs of HC	40.5%	13.6%	<0.001	14.3%
**≥**2 cognitive tests below −1.5 SDs of HC	27.0%	3.4%	<0.001	3.9%
**≥**1 cognitive test(s) below −2.0 SDs of HC	37.8%	11.9%	<0.001	10.9%
Below −1.5 SDs of HC mean, (*n*, %)			*p*‐values^a^	
Multitasking (MTT)	4 (5.4%)	11 (9.3%)	NS	
Memory (PAL)	18 (24.3%)	7 (5.9%)	0.005	
Response speed (RTI)	17 (23.0%)	9 (7.6%)	0.020	
Executive function (SWM)	15 (20.3%)	6 (5.1%)	0.023	
Attention (RVP)	18 (24.3%)	10 (8.5%)	0.041	
Below −2.0 SDs of HC mean, (*n*, %)			*p*‐values^a^	
Multitasking (MTT)	2 (2.7%)	3 (2.5%)	NS	
Memory (PAL)	11 (14.9%)	5 (4.2%)	0.044	
Response speed (RTI)	7 (9.5%)	2 (1.7%)	NS	
Executive function (SWM)	2 (2.7%)	2 (1.7%)	NS	
Attention (RVP)	11 (14.9%)	3 (2.5%)	0.021	
Subjective cognitive function (FACT‐Cog v3)			*p*‐values^a^	
Total score, mean (SD)	129.9 (19.5)	127.0 (17.3)	<0.001	
Perceived cognitive impairments (PCI), mean (SD)	73.0 (9.9)	68.8 (8.9)	<0.001	
Impact on quality of life (QOL), mean (SD)	11.9 (4.4)	14.0 (3.1)	0.039	
Comments from others (OTH), mean (SD)	15.4 (1.6)	15.3 (1.4)	0.048	
Perceived cognitive abilities (PCA), mean (SD)	29.6 (8.7)	28.8 (6.9)	NS	

*Note*: ^@^ Modeled based on Ingraham et al.^21^

Abbreviations: AYAC, AYA patients with cancer; HC, healthy control; SD, Standard Deviation; NS, not statistically significant.

^a^ Adjusted *p* values (adjusted for gender, age, ethnicity, marital status, highest education level, fatigue, and psychological distress).

A sensitivity analysis using propensity score methods demonstrated that these findings remained consistent after controlling for differences in baseline demographic characteristics **(**Data S2 **).**

When we evaluated individual cognitive tests, patients demonstrated more impairment (below −1.5 SD of HC mean) with memory (PAL), slower reaction times (RTI), strategy use (SWM) and attention (RVP) in the AYAC group compared to HC (all *p* values <0.05). (Table 1).

### 3.3 Self‐perceived cognitive function

AYAC self‐perceived less degree of cognitive impairment than HC (Table 3) (*p* < 0.001). However, AYAC perceived a greater impact of cognitive changes on their quality of life compared to HC. (*p* = 0.039) Both groups reported similar self‐perceived cognitive abilities.

There was no significant correlation between most of the subjective and objective domains, with the exception for the attention domain using CANTAB (RVP) and the subjective impact of cognitive problems on quality of life using FACT‐Cog (Rho = 0.203, *p* = 0.005) (Table S2).

### 3.4 Quality of life, symptom burden and cancer‐related fatigue

AYAC reported worse functioning in the physical, emotional, and school/work domains compared to HC (all *p* < 0.005). Both arms reported similar functioning for the social domain **(**Table 4 **)** AYAC reported significantly higher level of physical symptom distress, psychological distress, and higher impairment in activity level (*p* < 0.001).

**TABLE 4:** Mean baseline scores of PedsQL, RSCL and MFSI‐SF between AYAC and HC

	Patients (*N* = 74)	HC (*N* = 118)	*p*‐values
Quality of life (PedsQL^a^, mean [SD])			
Physical functioning	79.9 (21.4)	91.8 (11.7)	<0.001
Emotional functioning	64.2 (20.2)	78.1 (20.1)	<0.001
Social functioning	92.0 (12.7)	90.4 (12.8)	NS
School/Work functioning	79.2 (17.5)	84.6 (15.7)	0.03
Symptom burden (RSCL^b^, mean [SD])			
Physical symptom distress	13.2 (11.8)	8.5 (7.4)	<0.001
Psychological distress	30.8 (18.8)	17.2 (16.3)	<0.001
Activity level impairment	5.5 (10.4)	0.07 (0.5)	<0.001
Overall valuation of life	24.5 (17.7)	16.5 (16.4)	0.002
Fatigue (MFSI‐SF^c^, mean [SD])			
Total MFSI‐SF score	6.4 (16.4)	−1.2 (16.0)	0.002
General fatigue	5.3 (4.2)	4.9 (4.4)	NS
Physical fatigue	3.5 (3.6)	2.3 (3.0)	0.02
Emotional fatigue	7.1 (4.6)	3.3 (3.7)	<0.001
Mental fatigue	3.0 (3.6)	3.2 (3.7)	NS
Vigor	12.8 (5.5)	14.9 (4.9)	0.007

Abbreviations: AYAC, AYA patients with cancer; HC, healthy control; NS, not statistically significant.

^a^ A higher score with RSCL indicates higher level of burden or impairment.

^b^ A higher score with MFS‐SF indicates greater extent of fatigue.

^c^ A lower score with PedsQL indicates worse health‐related quality of life.

AYAC reported more fatigue compared to HC, based on total MFSI‐SF scores (*p* = 0.002), especially in the domain of emotional fatigue (*p* < 0.001).

### 3.5 Inflammatory cytokines

Blood samples from 60 AYAC and 118 HC were available for analysis. Elevations of baseline IL‐2 (*p* = 0.020), IL‐4 (*p* = 0.003), IL‐6 (*p* < 0.0001), IL‐8 (*p* < 0.0001), IL‐10 (*p* = 0.003) and IFN‐γ (*p* < 0.0001) were observed among AYAC versus HC (Table 5).

**TABLE 5:** Comparison of median plasma cytokine and BDNF levels between AYAC and HC

Biomarkers	Median levels (interquartile range) – AYAC	Median levels (interquartile range) – HC	Mann–Whitney U test *p* value
IL‐2 (pg/ml)	0.00 (0.00–1.36)	0.00 (0.00–0.20)	0.020
IL‐4 (pg/ml)	0.00 (0.00–0.64)	0.00 (0.00–0.00)	0.003
IL‐6 (pg/ml)	2.01 (0.96–3.57)	0.52 (0.00–1.06)	<0.001
IL‐8 (pg/ml)	5.86 (3.55–12.74)	4.13 (2.55–5.73)	<0.001
IL‐10 (pg/ml)	0.53 (0.00–1.65)	0.00 (0.00–0.53)	0.003
TNF‐α (pg/ml)	11.34 (6.87–19.93)	9.10 (7.00–12.02)	NS
GM‐CSF (pg/ml)	0.00 (0.00–0.27)	0.00 (0.00–0.00)	NS
IFN‐γ (pg/ml)	0.80 (0.37–1.58)	0.33 (0.00–0.59)	<0.001
BDNF (ng/ml)	10.74 (7.13–15.81)	21.60 (15.61–28.82)	<0.001

Abbreviations: AYAC, AYA patients with cancer; HC, healthy control; NS, not statistically significant.

Associations between IL‐2 and executive function (*p* = 0.038), IL‐6 and FACT‐Cog total score (*p* = 0.012), and TNF‐α and FACT‐Cog total score (*p* = 0.011) were observed among AYAC, whereas associations between IL‐2 and executive function (*p* = 0.001), GM‐CSF and response speed (*p* = 0.008) and TNF‐α and FACT‐Cog total score (*p* = 0.046) were observed among HC. **(**Table 6 **).**

**TABLE 6:** Association of biomarkers (cytokines and BDNF) with cognitive outcomes in AYAC and healthy controls

	Statistically significant cognitive outcomes	
Cytokines	AYAC	HC
IL‐2	Executive Function (*p* = 0.038)	Executive Function (*p* = 0.001)
IL‐4	NS	NS
IL‐6	FACT‐Cog Total Score (*p* = 0.012)	NS
IL‐8	NS	NS
IL‐10	NS	NS
GM‐CSF	NS	Response Speed (*p* = 0.008)
IFN‐γ	NS	NS
TNF‐α	FACT‐Cog Total Score (*p* = 0.011)	FACT‐Cog Total Score (*p* = 0.046)
BDNF	NS	NS

Abbreviation: NS, Not statistically significant.

### 3.6 BDNF levels

Blood samples for 60 AYAC and 117 HC were available for analysis. As opposed to cytokine levels, AYAC had significantly reduced expression of BDNF (*p* < 0.0001) compared to HC (Table 4). After controlling for factors mentioned, there was a lack of association observed between BDNF level and cognitive outcomes compared between AYAC and HC at baseline.

## 4 DISCUSSION

We have observed that pre‐treatment cognitive function was worse in newly diagnosed AYAC compared to age‐matched HC. Specifically, we observed that AYAC patients were 3 times more likely to perform poorly in at least more than 2 cognitive tests compared to HC. In contrast, self‐perceived cognitive impairment was more common among HC compared to age‐matched cancer patients. We also observed higher levels of baseline inflammatory markers and lower levels of baseline BDNF among AYAC in comparison to HC. Decreased levels of functioning, symptom burden, and fatigue levels among patients may have also contributed to the development of CRCI. Although AYAC may demonstrate greater resilience against cognitive impairment compared to older patients,^7^ our results suggest that physiological changes that are caused by cancer (such as inflammation) can predispose AYAC to subtle cognitive changes that may have gone unnoticed.

Human brains evolve continuously,^22^, ^23^ and physiological changes associated with the brain and age can be reflected by morphological (e.g., cortical thickness), biochemical (e.g., neuroinflammation), and behavioral changes (e.g., psychological distress).^24^, ^25^ Our observation of poorer objective cognitive function among newly diagnosed AYAC compared to HC prior to the initiation of cancer treatment is presumably due to alterations in inflammatory pathways, related to their cancer.^26^ AYAC also reported higher psychological distress, likely due to learning of a cancer diagnosis, which has been found to affect cognitive function. Similar trends have been observed among older patients with colorectal cancer where the domains of verbal learning, memory and processing speed were found to be more impaired than in HC.^12^

It was surprising that compared to HC, AYAC self‐perceived less cognitive impairment and reported less comments from others on changes in their cognitive function. Self‐perceived cognitive changes are known to be more associated with physical and psychological symptoms including fatigue, depression, and anxiety, which were more prominent among AYAC. However, differences noted between AYAC and HC were small and are not clinically important.

Our study has evaluated the role of neuroinflammation, specifically the link between inflammatory cytokines and cognitive function, associated with AYAC prior to cancer treatment. Inflammation had been identified as one of the hallmarks of cancer, with cytokines, chemokines, and growth factors shown to populate the tumor immune microenvironment.^27^ Peripheral inflammatory cytokines have been reported to trigger an inflammatory response in the brain, resulting in elevated oxidative stress.^28^ The younger age profile of our study cohort may have implications for the tumor microenvironment. Cytokine levels may exert greater influence on functional connectivity, relating to cortical thickness and surface area of the brain in adolescents.^29^ Age‐related immune differences have been reported, with lower IFN‐ƴ responses and lymphocyte infiltration observed in immune gene signatures of young adults with cancer.^30^ It is plausible that the cancer itself is sufficient to induce an inflammatory state, leading to the associations observed in this study between inflammatory biomarkers with response speed and executive function.

We observed that mean plasma BDNF levels in AYAC were less than half the levels among HC. Similar trends have been reported in studies involving older patients, although the observed differences were smaller.^31^, ^32^, ^33^, ^34^ This larger‐than‐expected difference could contribute to the higher degree of psychological distress in AYAC than HC, which has been linked with lower BDNF levels.^35^, ^36^ Physical activity levels are also related to higher BDNF expression,^37^ and newly diagnosed AYAC suffered from cancer‐related fatigue as well as limited by cancer‐induced mobility, which can impact the level of BDNF. BDNF is known to play an important role in neurogenesis and neuronal plasticity^38^, ^39^; hence, we postulate that BDNF might play a role in preserving cognitive function during chemotherapy.

There are several limitations in our study. The cohort consists of cancer patients diagnosed with 10 cancer types that are prevalent in the AYAC population. Knowing that cancer diagnosis is extremely very rare in terms of its incident between 16–39 years old, and our study was designed to address the understudied issue (such as prevalence) of CRCI among AYAC, our eligibility criteria were strategically designed to be based on age rather than cancer type or pathology. We acknowledge that this may threaten the external validity of our findings, but it would also be challenging to study the prevalence of CRCI in only one specific type of cancer among AYAC in view of its very rare incident.^5^ Investigating a specific disease subtype would also not be practical to generalize the knowledge to the entire AYA population. In view of the exploratory nature of our biomarker analysis, the analysis was not adjusted for multiple testing. Strengths of our study include incorporation of an age‐matched non‐cancer control group and evaluation of both subjective and objective cognitive performance, as well as a sensitivity analysis to confirm our primary findings. We have also incorporated AYAC‐specific and validated tools to assess and adjust for confounding factors.

## 5 CONCLUSION

Prior to cancer treatment, a higher proportion of AYAC experienced cognitive impairments on neuropsychological tests compared to HC; however, they were reporting less self‐perceived cognitive changes compared to HC. Biomarker analysis reported higher levels of neuroinflammation and lower levels of neurotrophin in AYAC, suggesting that AYA patients newly diagnosed with cancer are at high risks of developing CRCI even prior to the receipt of systemic treatment. With the pre‐existence of CRCI even prior to systemic therapy, we highly recommend clinicians to closely monitor the cognitive performance of AYA patients who are newly diagnosed with cancer. Further work should also be conducted to evaluate if earlier implementation of rehabilitation strategies to prevent cognitive deterioration will be beneficial for AYAC.

## AUTHOR CONTRIBUTIONS

**Alexandre Chan:** Conceptualization (lead); formal analysis (equal); funding acquisition (lead); investigation (lead); methodology (equal); project administration (equal); supervision (lead); writing – original draft (equal); writing – review and editing (equal). **Ivy Cheng:** Data curation (equal); formal analysis (equal); investigation (equal); methodology (equal); writing – original draft (equal); writing – review and editing (equal). **Claire Wang:** Data curation (equal); formal analysis (equal); investigation (equal); methodology (equal); writing – original draft (equal); writing – review and editing (equal). **Chia Jie Tan:** Data curation (equal); formal analysis (equal); investigation (equal); methodology (equal); writing – original draft (equal); writing – review and editing (equal). **Yi Long Toh:** Data curation (equal); formal analysis (equal); investigation (equal); methodology (equal); writing – original draft (equal); writing – review and editing (equal). **Ding Quan Ng:** Data curation (equal); formal analysis (equal); investigation (equal); methodology (equal); writing – original draft (equal); writing – review and editing (equal). **Yong Qin Koh:** Data curation (equal); formal analysis (equal); investigation (equal); methodology (equal); writing – original draft (equal); writing – review and editing (equal). **Hanzhang Zhou:** Data curation (equal); formal analysis (equal); investigation (equal); methodology (equal); writing – original draft (equal); writing – review and editing (equal). **Koon Mian Foo:** Conceptualization (equal); funding acquisition (equal); investigation (equal); project administration (equal); resources (equal); writing – original draft (equal); writing – review and editing (equal). **Raymond Javan Chan:** Conceptualization (equal); funding acquisition (equal); investigation (equal); project administration (equal); resources (equal); writing – original draft (equal); writing – review and editing (equal). **Han Kiat Ho:** Conceptualization (equal); funding acquisition (equal); investigation (equal); project administration (equal); resources (equal); writing – original draft (equal); writing – review and editing (equal). **Lita Chew:** Conceptualization (equal); funding acquisition (equal); investigation (equal); project administration (equal); resources (equal); writing – original draft (equal); writing – review and editing (equal). **Mohamad Farid:** Conceptualization (equal); funding acquisition (equal); investigation (equal); project administration (equal); resources (equal); writing – original draft (equal); writing – review and editing (equal). **Ian Tannock:** Conceptualization (equal); funding acquisition (equal); investigation (equal); project administration (equal); resources (equal); writing – original draft (equal); writing – review and editing (equal).

## CONFLICT OF INTEREST

The authors have declared no competing interest.

## Supporting information


Table S1
Table S2

## Data Availability

The data that support the findings of this study are available in the Supplementary material of this article.
