# Supplementary material for: Cognitive impairment in adolescent and young adult cancer patients: Pre‐treatment findings of a longitudinal study
Source: Cancer Med. 2022 Oct 11;12(4):4821–31. doi: 10.1002/cam4.5295 (PMC9972136; doi:10.1002/cam4.5295)
Supplement: Supplementary file 1 — Table S1 Table S2 [file CAM4-12-4821-s001.docx]

**Supplementary Table 1: Individual domain raw scores for each cognitive test**

| Individual domain raw scores |  |  | *p*-values^#^ |  |
| --- | --- | --- | --- | --- |
| Multitasking (MTT), mean (SD) | 203.5 (108.8) | 196.8 (112.7) | NS |  |
| Memory (PAL), mean (SD) | 13.0 (11.7) | 8.1 (8.0) | NS |  |
| Response speed (RTI, ms), mean (SD) | 397.7 (40.0) | 370 (36.5) | **0.001** |  |
| Executive function (SWM), mean (SD) | 7.9 (2.9) | 7.3 (2.3) | NS |  |
| Attention (RVP), mean (SD) | 0.9 (0.05) | 0.9 (0.04) | NS |  |

**Supplementary Table 2: Correlations between pre-treatment objective and subjective cognitive function at baseline**

|  |  | **Objective (CANTAB®)** | | | | |
| --- | --- | --- | --- | --- | --- | --- |
|  | Rho (*p*-value) | **Multitasking** | **Memory** | **Response speed** | **Executive function** | **Attention** |
| **Subjective (FACT-Cog)** | **Perceived cognitive impairments** | 0.094  (0.192) | 0.076  (0.296) | 0.098  (0.175) | 0.126  (0.081) | 0.012  (0.864) |
|  | **Impact on quality of life** | 0.048  (0.510) | -0.093  (0.202) | -0.131  (0.071) | -0.64  (0.380) | 0.203*  (0.005) |
|  | **Comments from others** | 0.135  (0.063) | -0.87  (0.232) | 0.099  (0.171) | 0.069  (0.345) | 0.059  (0.418) |
|  | **Perceived cognitive abilities** | 0.085  (0.241) | -0.027  (0.714) | 0.134  (0.064) | 0.059  (0.418) | 0.083  (0.253) |
|  | **Overall subjective cognitive function** | 0.078  (0.285) | -0.009  (0.899) | 0.069  (0.342) | 0.046  (0.530) | 0.111  (0.126) |

* *p* < 0.05

**Supplementary Material 1: Description of key measures for quantifying individual cognitive domain scores**

| Cognitive domains | Key measure description |
| --- | --- |
| Multitasking (MTT) | Multitasking cost (median): The difference between the median latency of response (from stimulus appearance to button press) during assessed blocks in which both rules are used versus assessed blocks in which only a single rule is used. Calculated by subtracting the median latency of response during single task block(s) from the median latency of response during multitasking block(s). A positive score indicates that the subject responds more slowly during multitasking blocks, and indicates a higher cost of managing multiple sources of information. |
| Memory (PAL) | PAL Total Errors (Adjusted): The number of times the subject chose the incorrect box for a stimulus on assessment problems (PALTE), plus an adjustment for the estimated number of errors they would have made on any problems, attempts and recalls they did not reach. This measure allows you to compare performance on errors made across all subjects regardless of those who terminated early versus those completing the final stage of the task. In this task variant PALTEA does not include 12 box level to provide a direct comparison to Recommended Standard. |
| Response speed (RTI) | RTI Median Five-Choice Reaction Time: The median duration it took for a subject to release the response button after the presentation of a target stimulus. Calculated across correct, assessed trials in which the stimulus could appear in any one of five locations. Measured in milliseconds. |
| Executive function (SWM) | SWM Strategy (6-8 boxes): The number of times a subject begins a new search pattern from the same box they started with previously. If they always begin a search from the same starting point we infer that the subject is employing a planned strategy for finding the tokens. Therefore a low score indicates high strategy use (1 = they always begin the search from the same box), a high score indicates that they are beginning their searches from many different boxes. Calculated across assessed trials with 6 tokens or 8 tokens. |
| Attention (RVP) | RVP A prime (A’): A' is the signal detection measure of sensitivity to the target, regardless of response tendency (expected range 0.000 to 1.000; bad to good). In essence, this metric is a measure of how good the subject is at detecting target sequences. |

**Supplementary Material 2: Propensity score weighting**

In our sensitivity analysis, propensity score weighting using inverse-probability weights was carried out to balance the baseline demographics of adolescent and young adult cancer patients (AYAC) and healthy controls (HC)^1,2^. Using this approach instead of other propensity score methods allowed us to retain all study subjects in our analysis. The propensity score model included the covariates age, gender, race and marital status (where never married, divorced and widowed were collectively considered as unpartnered) and years of education and was constructed using logistic regression with AYAC or HC membership serving as the dependent variable. Acceptable goodness-of-fit was demonstrated using the Hosmer-Lemeshow test (p = 0.69) while baseline covariates were balanced after propensity score weighting with standardized differences less than 0.2 and variance ratios close to 1.0 (Table SM1) ^3^. The assumptions of positivity and overlap was checked by visually assessing the distribution of propensity scores^3^. No observations with extreme propensity scores (<1 x 10^-5^ or > 1 - 1 x 10^-5^) were noted^3^.

The prevalence of cognitive impairment in each group estimated in our sensitivity analysis was consistent with that in the primary analysis with differences of not more than 5%. Differences in prevalence between AYAC and HC remained statistically significant (Table SM2).

**Table SM1 Diagnostics for propensity score model**

| Variables | Unweighted | | Weighted | |
| --- | --- | --- | --- | --- |
|  | Standard difference | Variance  ratio | Standard difference | Variance  ratio |
| Age | 0.28 | 0.85 | 0.15 | 1.42 |
| Gender: Male | 0.00 | 1.00 | 0.12 | 1.08 |
| Gender: Female | 0.00 | 1.00 | 0.12 | 1.08 |
| Race: Chinese | 0.15 | 0.87 | 0.01 | 0.99 |
| Race: Malay | 0.56 | 0.12 | 0.10 | 0.69 |
| Race: Indian | 0.42 | 2.97 | 0.04 | 1.08 |
| Race: Others | 0.16 | 0.54 | 0.04 | 1.11 |
| Marital status: Married | 0.38 | 1.00 | 0.14 | 1.00 |
| Marital status: Unpartnered | 0.38 | 1.00 | 0.14 | 1.00 |
| Years of education | 0.89 | 0.82 | 0.05 | 0.79 |

**Table SM2 Results of sensitivity analysis**

| Objective cognitive function (CANTAB®) | AYAC (%)  (N = 74) | HC (%)  (N = 118) | *p-values* |
| --- | --- | --- | --- |
| ≥ 2 cognitive tests below -1.5 SDs of HC or ≥ 1 cognitive test(s) below -2.0 SDs of HC | 42.8% | 14.9% | **0.0014** |
| ≥ 2 cognitive tests below -1.5 SDs of HC | 22.6% | 3.3% | **0.0011** |
| ≥ 1 cognitive test(s) below -2.0 SDs of HC | 31.2% | 12.8% | **0.0011** |

AYAC = AYA patients with cancer; HC=healthy control

References:

1. Austin PC. An Introduction to Propensity Score Methods for Reducing the Effects of Confounding in Observational Studies. *Multivariate Behav Res*. 2011;46(3):399-424. doi:10.1080/00273171.2011.568786

2. Beal SJ, Kupzyk KA. An Introduction to Propensity Scores. *J Early Adolesc*. 2014;34(1):66-92. doi:10.1177/0272431613503215

3. Austin PC, Stuart EA. Moving towards best practice when using inverse probability of treatment weighting (IPTW) using the propensity score to estimate causal treatment effects in observational studies. *Stat Med*. 2015;34(28):3661-3679. doi:10.1002/sim.6607
